# Professional Quality of Life among Medical Doctors Working in Kathmandu: A Descriptive Cross-sectional Study

**DOI:** 10.31729/jnma.5330

**Published:** 2020-11-30

**Authors:** Anju Vaidya, Shristi Karki, Meghnath Dhimal, Pradip Gyanwali, Dibash Baral, Ashok Pandey, Anjani Kumar Jha

**Affiliations:** 1 Nepal Health Research Council, Ramshah Path, Kathmandu, Nepal; 2 Public Health Promotion and Development Organization, Chandole, Kathmandu, Nepal

**Keywords:** *burnout*, *compassion satisfaction*, *doctors*, *secondary traumatic stress*

## Abstract

**Introduction:**

The practice of medicine is an honorable profession besides being accompanied by a demanding environment. This study aimed to find out the professional quality of life of medical doctors working in Kathmandu valley.

**Methods:**

A descriptive cross-sectional study was conducted among 174 Nepalese medical doctors working in different hospitals of Kathmandu valley. Ethical approval was taken from the Ethical Review Board of the Nepal Health Research Council (Reference Number: 830). The data collection tool used in the study was WHO Professional Quality of Life Scale-5 to collect data about Compassion satisfaction, Burnout and Secondary traumatic stress among medical doctors working in Kathmandu valley. Data analysis was done in the Statistical Package for the Social Sciences version 16.0.

**Results:**

Out of 174 participants, 101 (58%), 126 (72.4%) and 135 (77.6%) were found to have moderate level of Compassion satisfaction, Burnout and Secondary Traumatic Stress respectively.

**Conclusions:**

More than half, nearly two-third, and more than two-third participants had moderate levels of Compassion satisfaction, Burnout and Secondary Traumatic Stress respectively. The overall study findings reflected good balance between Compassion satisfaction and Compassion fatigue (burnout and secondary traumatic stress) among the Nepalese medical doctors. Further assessment of professional quality of life of doctors as well as other health care workers via Multifaceted and large-scale study is recommended.

## INTRODUCTION

Occupation is one of the important aspects of life. There is, therefore, no doubt that a person's point of view towards their work and experiences he has been through is framed by the nature of his work also known as Professional Quality Of Life (Pro QOL).^1^

Studies conducted in Asian countries reflect variations in its findings. Quality of life of doctors working in China, Bangladesh and Lahore was found to be low^2–4^ while the overall career satisfaction among Sri Lankan and Thai doctors was higher.^5,6^ In the context of Nepal, doctor per population ratio in 2014 was 0.6 per 1000 reflecting high demand of doctors.^7^ In addition to efforts that are being made to provide quality health care to the Nepalese population, the impetus to improve the quality of life of Nepalese doctors also requires priority.

The objective of the study is to find out the Pro QOL of medical doctors working in Kathmandu valley, Nepal.

## METHODS

This was a descriptive cross-sectional study. The target population for the study was Nepalese medical doctors working in different hospitals of Kathmandu valley. The study commenced after obtaining approval from the Ethical Review Board of Nepal Health Research Council (Reference Number: 830), Kathmandu, Nepal. The informed consent was obtained from the participants before data collection via email or in written form. Nepalese medical doctors from different specialties and of all levels who have been working for at least six months in Kathmandu valley were included in the study. The recruitment of the participants was done using a convenient sampling technique.

The sample size was calculated considering estimates from the previous study^8^ and using formula,

$$\begin{array}{ll} \text{n} & \text{= }{\text{Z}}^{\text{2}}\times \text{p}\times \text{(1}-\text{p)}/{\text{e}}^{\text{2}}\\ & \text{= }{\left(1.96\right)}^{\text{2}}\times (0.12)\times (1-0.12)/{(\text{0.05)}}^{\text{2}}\\ & \text{= }162.2\\ & =162 \end{array}$$

where,

n = sample size
Z = 1.96 at 95% Confidence Interval
p = prevalence, 12%^8^
q = 1-p
e = margin of error, 5%

The minimum sample size was calculated to be 162 considering 5% margin of error and 95% confidence interval.

The principal investigator, being herself a medical doctor, contacted her colleagues and friends working in hospitals, who volunteered and coordinated for data collection in the present study. A total of 200 medical doctors working in different departments and different public and private hospitals of Kathmandu valley, who met the inclusion criteria, were approached face to face or electronically. Of the 200 participants contacted, 174 participants gave consent to participate in the study via email or writing.

The data collection tool used in the study had two sections. First, sociodemographic questionnaire to collect information about sociodemographic details, professional details, and work-related information. Second, the Professional Quality of life Scale 5, which was used to collect data about Burnout, Compassion Satisfaction and Secondary traumatic Stress.^9^ Data was collected electronically using a self-administered semi-structured questionnaire. The electronic version of the questionnaire was sent to the physicians through email and was collected once filled completely. If the participants wished to fill the questionnaires manually, we provided them with the questionnaire in paper form.

The collected data was entered into Epidata Version 1.6.0. Data analysis was carried out using IBM SPSS Statistics for Windows, version 16.0. All the variables were checked for missing data and typo errors. Computation of Means, Standard Deviation (SD), frequency counts and percentages of all sociodemographic and professional data were done. Means, Standard Deviations and percentages of CS, BO and STS were computed.

## RESULTS

The mean age of the study population was 35.97±10.04 years, ranging from 25 to 65 years of age. The participants of the study were mostly men, 114 (65.5%), with the majority falling under the age of 40 years and below in 127 (77%). Different positions held by the participants in the current study were 59 (33.9%) medical officers, 22 (12.6%) registrars, 73 (42%) consultants and 20 (11.5%) residential medical officers. Fifty-four (31.1%) participants of the study had completed only MBBS, 76 (43.7%) participants had completed Doctor of Medicine (MD), 27 (15.5%) participants had completed MS, 7 (4%) participants had completed Diploma, while 10 (5.7%) participants had completed their last education in other fields such as Fellowship of the College of Physicians (FCPS), FRCOG, MCH, DM and other fellowships. Of the 174 participants, 29 (16.7%) had completed their specialization as a General physician, 18 (10.3%) in Orthopedics, 19 (10.9%) in Obstetrics and Gynecology, while 46 (26.4%) participants had completed their specialization in various other fields such as pediatrics, radiology, neurology, cardiology, etc. (Table 1).

**Table 1:** Socio-demographic and work-related characteristics.

Demographic variables	Frequency n (%)
Age (years) (n = 165)	
40 and below	127 (77)
Above 40	38 (22.1)
Gender (n = 174)	
Male	114 (65.5)
Female	60 (34.5)
Current position (n = 174)	
Medical Officer	59 (33.9)
Registrar	22 (12.6)
Consultant	73 (42.0)
Residential Medical Officer	20 (11.5)
Qualification (n = 174)	
MBBS (only)	54 (31.1)
Diploma	7 (4)
MD	76 (43.7)
MS	27 (15.5)
Others	10 (5.7)
Specialization (n = 174)	
General Physician	29 (16.7)
Specialization in	
Orthopedics	18 (10.3)
Obstetrics and Gynecology	19 (10.9)
Others	46 (26.4)
Years in field (n = 174)	
<5 years	78 (44.8)
5-15 years	63 (36.2)
>15 years	33 (19)
Annual Income (n = 174)	
Less than 5 lakhs/annum	85 (48.9)
5-10 lakhs/annum	42 (24.1)
10-15 lakhs/annum	23 (13.2)
More than 15 lakhs/annum	23 (13.2)
Practicing sector (n = 174)	
Public Sector	82 (47.1)
Private Sector	131 (75.3)
Both	40 (23)
Working hours in a day (n = 174)	
<=8 hours	91 (51.6)
>8 hours	83 (47.5)

Duration of working as a doctor in the medical field ranged from 6 months to 40 years (mean = 9.19±8.93 years). Among them, 78 (44.8%) participants had working experience in the field for less than 5 years, 63 (36.2%) participants and 33 (19%) participants had work experience in the field for 5-15 years and more than 15 years respectively. Similarly, 82 (47.1%) doctors and 131 (75.3%) doctors were working in public and private hospitals respectively, among whom 40 (23%) doctors were working in both sectors.

The mean number of days worked by the participants in the week was 5.74±1.52. Similarly, the average number of hours worked daily at the workplace to treat patients ranged from 3 to 16 hours (mean = 9.07±2.96). Eight (4.6%) out of 174 participants reported their average number of working hours per day as 24 hours which was equated to 16 hours. In a similar line, 91 (51.6%) participants were working for less than equal to 8 hours a day while 83 (47.5%) participants were working for more than 8 hours a day. Mean number of hours worked in a week was 53.19±24.09 hours. Mean cases seen per day was 27.27±19.54 which ranged from 2 to 100 depending on designation and workplace. Similarly, annual income of 85(48.9%), 42 (24.1%), 23 (13.2%) and 23 (13.2%) participants were less than 5 lakhs/annum, 5-10 lakhs/annum, 10-15 lakhs/annum and more than 15 lakhs/annum respectively.

The raw scores of CS, BO and STS was converted into t scores using the concise ProQOL manual by Stamm, 2010.^9^ The mean scores for the level of CS, BO and STS among medical doctors working in Kathmandu valley were 40.56±5.50, 24.85±4.74 and 27.70±6.23 respectively (Table 2). One hundred and one (58%), 126 (72.4%) and 135 (77.6%) medical doctors were found to have moderate levels of CS, BO and STS respectively. Interestingly, all the study population were found to have moderate 101 (58%) to high 73 (42%) level of CS with none having a lower level of CS. Likewise, BO among them ranged between low 48 (27.6%) and moderate 126 (72.4%) level while none of them had a higher level of BO. Similarly, the majority of the study population experienced lower 35 (20.1%) to moderate 135 (77.6%) level of STS while very few 4 (2.3%) experienced a higher level of STS. The relationship of CO, BO, and STS with sociodemographic and work-related characteristics is shown (Table 2).

**Table 2:** Compassion Satisfaction, Burnout and Secondary Traumatic Stress among medical doctors (n = 174).

Scale	^*^CS n (%)	BO^†^ n (%)	STS^‡^ n (%)
Low	0 (0)	48 (27.60)	35 (20.10)
Moderate	101 (58)	126 (72.4)	135 (77.60)
High	73 (42)	0 (0)	4 (2.30)
Mean	40.56	24.85	27.70
Std. Deviation	5.50	4.74	6.23

* CS: Compassion satisfaction;

† BO: Burnout;

‡ STS: Secondary Traumatic Stress.

## DISCUSSION

Pro QOL has become a growing field of interest along with the development of better tools in the context of health care professionals. Several studies done in both low-middle- and high-income countries depict interesting findings regarding QoL of health professionals. Studies done in developed countries such as Norway and United States indicates that burnout and low level of life satisfaction is highly prevalent among the physicians compared to the general population.^10,11^ Additionally, stressors affecting QoL begin with undergraduate medical education. The prevalence of psychological problems was found high with the stressors mainly related to academic and psychosocial factors among undergraduate medical students in Nepal.^12^ Moreover, there is a paucity of data to acknowledge the QoL of Nepalese physicians.

As per the NMC website, there are altogether 23147 doctors registered in Nepal Medical Council as of 31st December 2019.^13^ There is a high demand of doctors in Nepal where doctor per population ratio in 2014 was 0.6 per 1000.^7^ Limited number of doctors for proportionately a huge number of patients are more prone to having increased workload associated with an increased level of BO and dissatisfaction leading to higher chances of adversely affecting healthcare system and service delivery.

The results of this study depicted the majority of medical doctors having an average level of CS, BO and STS. These findings reflect a good balance between CS (positive factor) with Compassion Fatigue that includes Burn out and Secondary Traumatic Stress (negative factor) which motivates the medicals doctors to adjust in their working environment and be resilient. More than half of the population (58%) reported having average CS while nearly half of the study population (42%) reported having high CS which indicates that more than half of the study participants were positive about helping others and they derived pleasure from being able to do their work well. A similar study conducted among medical doctors of Sri-Lanka revealed findings in line with this study.^6^ This study also showed that as the doctors grew older, their work experience also increased along with their job satisfaction. The study findings were quite similar to a study conducted in India where a higher level of job satisfaction among doctors specially males, above 60 years of age, those working in the same institution for more than 5 years.^14^ This might be due to various reasons such as a decrease in expectations and adaptation to the situation. In contrast, doctors with young age increased expectations from their job resulting in job dissatisfaction.^15^ The study population experienced low to moderate BO in this study which indicates that the individuals are at risk. The results also indicate that they are unhappy, disconnected; and have a feeling of being bogged down and exhausted due to their working environment.^9^ The association of burnout in medical doctors and patient care has not been assessed in this study which is definitely a study in need but burnout was found to be associated with number of working hours per day. Studies conducted in different countries have shown varied findings in case of burnout among medical doctors. A nation-wide study conducted in China depicted 73.6% of medical doctors to have very high Burnout which contrasts this study finding.^16^

Meanwhile, there is a paucity of information related to burnout in the context of Nepalese doctors. There is a strong need for more extensive and representative research focusing on the professional quality of life of medical professionals. More than two-third of the participants (77.6%) reported having a moderate level of Secondary traumatic stress which is related to vicarious trauma.^9^

In contrast to these findings, studies conducted in both developed and developing countries such as the USA and India showed high prevalence of secondary traumatic stress among doctors.^17,18^ This reflects better professional quality of life of doctors in Nepal with Compassion fatigue low to moderate. Since this study was conducted among medical doctors who practiced only in Kathmandu valley, further exploration of these factors in other parts of the country is indicated. In addition, further exploration of these outcome variables among other health care professionals like nurses, paramedics, etc. is highly recommended as only medical doctors are enrolled in this study.

## CONCLUSIONS

This study reflects balance between positive and negative factors of Pro QOL among Nepalese doctors that is moderate to high compassion satisfaction, low to moderate burnout and secondary traumatic stress. Taking into consideration the limitation of this study to Kathmandu valley and enrolment of only doctors, a multifaceted large-scale study can put further insight on Pro QOL of health professionals. Further exploration of ProQOL of health professionals and quality of care provided by them are equally important paving the way forward towards more comprehensive studies and addressing the issues related to their QoL.
